# Wilson disease associated with a novel variant in the *ATP7B* gene

**DOI:** 10.3389/fneur.2026.1855936

**Published:** 2026-08-10

**Authors:** Lipeng Yang, Jun Li, Si Xie, Xinhong Feng

**Affiliations:** 1 Department of Neurology, Beijing Tsinghua Changgung Hospital, School of Clinical Medicine, Tsinghua Medicine, Tsinghua University, Beijing, China; 2 Community Health Service Center, Beijing Normal University, Beijing, China; 3 Department of Hepatobiliary Medicine, Beijing Tsinghua Changgung Hospital, School of Clinical Medicine, Tsinghua Medicine, Tsinghua University, Beijing, China

**Keywords:** ATP7B, case, compound heterozygous mutation, gene mutation, Wilson disease

## Abstract

**Introduction:**

Wilson disease is an autosomal recessive monogenic disorder caused by mutations in the copper-transporting P-type ATPase beta gene (*ATP7B*) located on human chromosome 13. This gene encodes copper-transporting P-type ATPase. This article reports a case of Wilson disease with a novel *ATP7B* deletion variant.

**Case report:**

We report a case of Wilson disease in a Chinese female patient. Cirrhosis was detected during routine examination at the age of 24 and was accompanied by reduced serum ceruloplasmin levels. At the age of 27, she gradually developed tremors and dystonia. Brain magnetic resonance imaging (MRI) revealed abnormal signals in the basal ganglia. Genetic testing for *ATP7B* was performed on the patient and her family members.

**Results:**

Genetic testing revealed that the patient harbored compound heterozygous variants, including a previously reported c.1543+40G>A variant and a novel c.837delT variant in exon 2, which has not been previously reported. The c.1543+40G>A variant is registered in the ClinVar database with conflicting interpretations of pathogenicity (likely pathogenic/likely benign), The c.837delT variant is predicted to result in a frameshift (p.Ile279Metfs*5), introducing a premature termination codon, and is thus likely to impair the function of the copper-transporting P-type ATPase.

**Conclusion:**

We identified a novel deletion variant in a Wilson disease patient harboring compound heterozygous variants in *ATP7B*. This finding expands the known spectrum of pathogenic *ATP7B* variants.

## 1 Introduction

Wilson disease is a rare, inherited, progressive disorder that severely impairs copper metabolism (1). It can manifest at any age, with a higher incidence between the ages of 5 and 35. It can affect multiple organs and lead to complex clinical presentations and diverse symptoms. When the liver is involved, clinical manifestations primarily include nausea, fatigue, and jaundice. Cirrhosis or liver failure may occur (2). When the central nervous system is affected, patients often exhibit symptoms, such as behavioral abnormalities, personality changes, and ataxia (3).

Wilson disease is closely associated with the ATPase copper transporting beta (*ATP7B*) gene, which primarily encodes a P-type ATPase. The encoded protein specifically binds to ceruloplasmin precursor to form ceruloplasmin. *ATP7B* is an established causative gene of Wilson disease. According to the Human Genome Organisation (HUGO) database, over 700 pathogenic variants have been reported in *ATP7B.* Among these, single-nucleotide missense and nonsense variants are the most common, followed by insertion/deletion and splice-site variants (4, 5). These variants are distributed across all exons and introns^1^ (6, 7).

We report a case of a young female patient who initially presented with abnormal liver function and subsequently developed progressive neurological symptoms. Genetic analysis revealed that she carried a c.1543+40G>A intronic variant in *ATP7B*, which was inherited from her heterozygous mother, and a novel c.837delT deletion variant in exon 2, which has not been previously reported.

## 2 Case presentation

**Figure 1:**
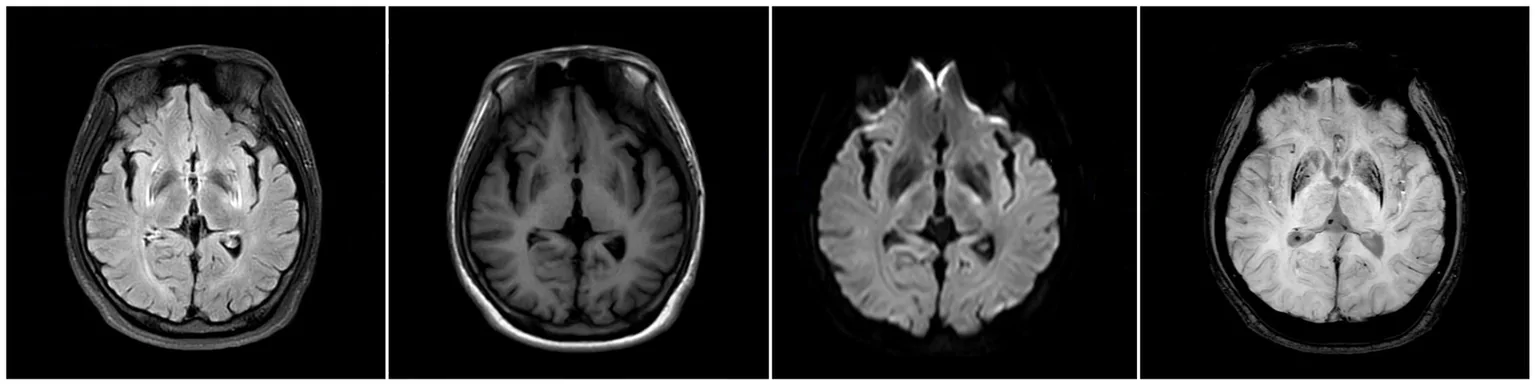
Brain MRI revealed abnormal signals in the bilateral putamen.

The proband was a young female with disease onset at 24 years of age. She was born to non-consanguineous parents, and her elder brother and sister were asymptomatic. The patient was married, and had no children. Abnormal liver function was initially detected during routine medical examination, which gradually progressed to liver cirrhosis. Liver ultrasound revealed reduced liver volume, irregular capsule, blunted edge, heterogeneous and decreased echogenicity of the liver parenchyma, clear vascular texture, no dilation of the intrahepatic or extrahepatic bile ducts, and diffuse hyperechoic nodule-like lesions within the liver. Contrast-enhanced upper abdominal computed tomography (CT) revealed liver cirrhosis and splenomegaly. The patient was regularly followed-up and treated for liver cirrhosis at another hospital and received oral hepatoprotective medications. Three years after the discovery of abnormal liver function, the patient developed involuntary tremors in her right lower limb, which became more pronounced during emotional stress or focused attention. She presented to the neurology clinic and underwent brain MRI, which revealed bilateral symmetric putaminal hypointensity on T1-weighted imaging and hyperintensity on T2-weighted imaging (Figure 1). Serum ceruloplasmin levels were measured at 0.04 g/L. The neurologist recommended hospitalization for a detailed evaluation and genetic testing. The patient denied any family history and refused further investigation. Six months later, her neurological symptoms progressively worsened, manifesting as slurred speech, orofacial dystonia, risus sardonicus, and abnormal postural reflexes. The patient was subsequently admitted to our hospital for treatment.

Neurological Examination: The patient was conscious of mild dysarthria and had normal cognitive function. Kayser–Fleischer rings were positive under slit-lamp examination. Orofacial dystonia was also observed. The muscle strength in all four limbs was normal. Coordination was intact. Bilateral dysdiadochokinesia was observed during finger-to-thumb opposition and foot tapping, and the pull test results were positive. Postural tremors were observed in the right limb. Sensory examination results were normal, and pathological reflexes were negative.

The laboratory findings revealed the following: blood copper level was 193.1 μg/L; 24-h urinary copper excretion was 88.2 μg/24 h; serum ceruloplasmin was 0.08 g/L; blood ammonia was 84.7 μmol/L. Liver function tests showed alanine aminotransferase (ALT) at 20.4 U/L, aspartate aminotransferase (AST) at 29.1 U/L, alkaline phosphatase (ALP) at 74 U/L, and gamma-glutamyl transferase (GGT) at 27 U/L. Bilirubin levels included total bilirubin (TBIL) 22.3 μmol/L and direct bilirubin (DBIL) of 9.9 μmol/L.

## 3 Methods

Genomic DNA was extracted from peripheral blood samples. The proband was tested using an *ATP7B*-targeted gene panel. Raw reads were aligned to the human reference genome (hg19) using the Burrows-Wheeler Transform (BWT) algorithm. Candidate variants were then annotated with information from multiple databases, including the Online Mendelian Inheritance in Man (OMIM), ClinVar, Decipher, the Database of Genomic Variants (DGV), the Human Phenotype Ontology (HPO) database, population frequency databases, and bioinformatics prediction tools. Sanger sequencing was performed to validate the identified variants in the proband and her family members. The sequencing results were compared with the reference cDNA sequence (NM_000053).

## 4 Results

### 4.1 Clinical analysis

We conducted a study of a family in which one member was diagnosed with Wilson disease. The proband presented abnormal liver function, liver cirrhosis, and neurological and psychiatric symptoms. According to the Leipzig scoring system, the patient scored 10 points (2 points for Kayser–Fleischer rings, 2 points for neurological symptoms, 2 points for ceruloplasmin level, and 4 points for genetic testing), meeting the diagnostic criteria for Wilson disease. The parents, one elder brother, and one elder sister all had normal liver function and neurological examination findings, as well as normal ceruloplasmin levels.

### 4.2 Genetic testing results and verification

Compound heterozygous variants were identified in the proband, including a previously reported c.1543+40G>A variant and a novel c.837delT variant in exon 2, which has not been previously documented. The c.1543+40G>A variant is currently registered in ClinVar (Accession: VCV003576314.4) with conflicting interpretations of pathogenicity, including three submissions of likely pathogenic and one of likely benign. The c.837delT variant is predicted to cause a frameshift, p.Ile279Metfs*5, resulting in a premature termination codon five residues downstream. This variant has not been previously reported and is not listed in ClinVar or HGMD databases.

**Figure 2:**
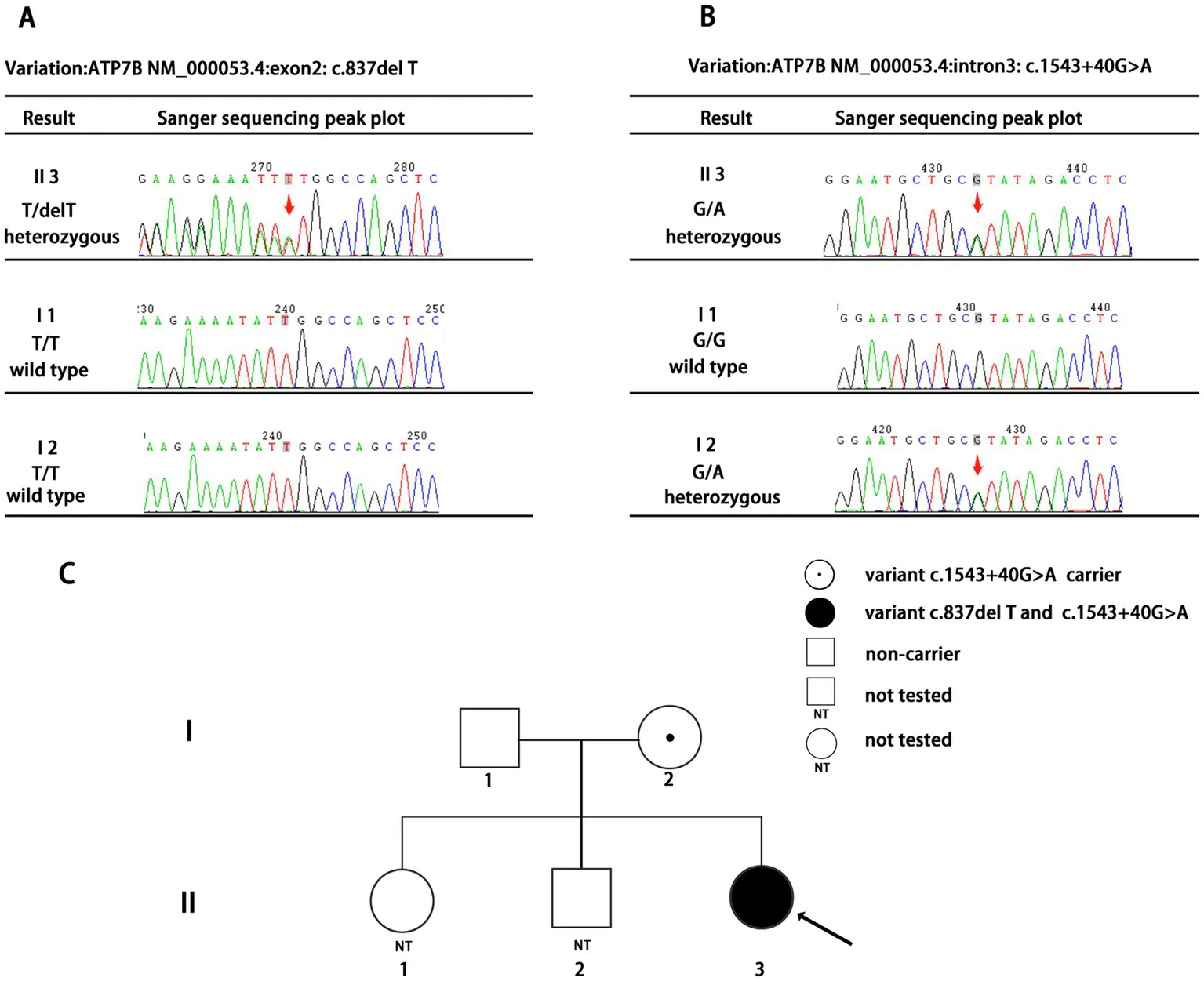
**(A)** Sanger sequencing verification of the *ATP7B* c.837delT variant in the proband’s parents, both of whom tested negative for this variant **(B)** sanger sequencing verification of the *ATP7B* c.1543+40G>A variant in the proband’s parents, showing that the father tested negative while the mother was a heterozygous carrier **(C)** pedigree chart of the proband’s family, indicating that the mother carries the c.1543+40G>A variant, the father carries neither mutation, and the proband’s elder brother and elder sister were not genetically tested.

Sanger sequencing confirmed that the proband’s mother was a heterozygous carrier of the c.1543+40G>A variant, whereas the father had a normal genotype. Neither parent carried the novel c.837delT variant (Figure 2). Because the patient’s elder brother and sister lived in another province and declined to come to our city for genetic testing, unfortunately we were unable to obtain their genetic test results. However, they were asymptomatic and had normal ceruloplasmin levels.

## 5 Discussion

*ATP7B* is located on chromosome 13q14.3, spanning approximately 100 kb, and consists of 21 exons and 20 introns (8, 9). *ATP7B* belongs to the highly conserved type IB (PIB) subfamily of P-type ATPases, which are responsible for transporting copper and other heavy metals across cellular membranes (10). This protein comprises 1,465 amino acids and contains distinct structural domains, including a phosphatase domain (A-domain), a phosphorylation domain (P-domain, amino acid residues 971–1,035), a nucleotide-binding domain (N-domain, residues 1,240–1,291), and an M-domain formed by eight transmembrane helices that constitute the ion channel (11–14).

The core structure of each domain features unique amino acid motifs such as TGEA in the A domain, DKTGT in the P domain, and SEHPL in the N domain. The N-terminal metal-binding domain (MBD) consists of six copper-binding sites, each containing the conserved sequence motif GMXCXXC (15, 16). These MBDs play a central role in accepting copper from the copper chaperone ATOX1 through protein–protein interactions.

According to previous reports (17–19), the intronic variant c.1543+40G>A was identified in three patients with Wilson disease. Among them, two were compound heterozygotes, the c.3836A>G and c.2447+5G>T variants on the opposite alleles, respectively. The genetic status of the third patient was not specified. Xu et al. (18) performed splicing analyses using a minigene system and *in vivo* RT-PCR, both of which showed that c.1543+40G>A leads to skipping of exon 3. Therefore, although this variant is registered in the ClinVar database as conflicting interpretations of pathogenicity (likely pathogenic/likely benign), based on the above discussion, we consider the variant to be more likely pathogenic.

**Figure 3:**
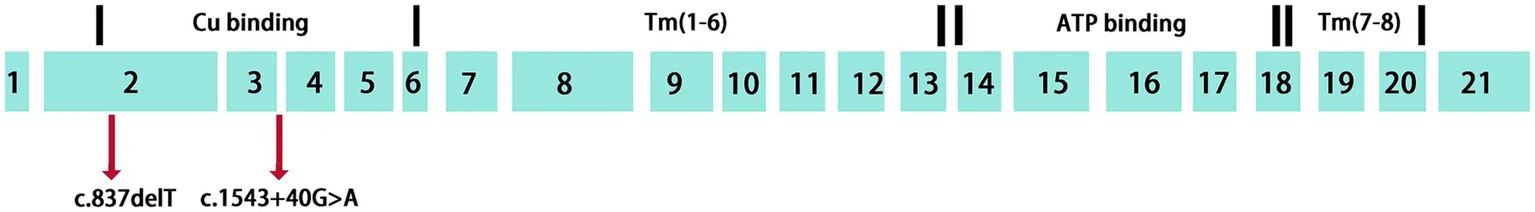
The locations of the proband’s compound heterozygous variants are marked on the schematic diagram of the *ATP7B* gene.

The novel *ATP7B* variant c.837delT identified in this patient caused a frameshift variant (p.Ile279Metfs*5), introducing a premature termination codon at the fifth downstream codon. This results in the premature termination of *ATP7B* protein synthesis (Figure 3). Consequently, only the first three of the six conserved GMXCXXC repeats within the metal-binding domain are predicted to be retained in the truncated protein. Previous studies have indicated that the impact of individual MBDs on *ATP7B* activity is not equivalent, with MBDs 5 and 6 playing more critical roles in the catalytic activation of *ATP7B* than MBDs 1–4 (20). Based on the structural alterations described above, it can be inferred that this variant impairs the function of the copper-transporting P-type ATPase.

It is well established that loss-of-function of the ATP7B gene is the pathogenic mechanism underlying Wilson disease. The c.837delT variant leads to a frameshift mutation, which is predicted to result in loss of protein function; according to the ACMG/AMP criteria (21), this fulfills the PVS1 criterion (very strong). The PVS1 criterion addresses the classification of the variant itself rather than the allelic configuration. Although formal phasing was not performed, the definite clinical diagnosis of Wilson disease combined with the segregation data supports the trans configuration (compound heterozygosity) of these two variants underlying the recessive phenotype. This variant is absent from normal population databases (Exome Sequencing Project, 1,000 Genomes, and ExAC), meeting the PM2 criterion (moderate). Although the patient carries both c.837delT and c.1543+40G>A, we were unable to demonstrate that these two variants are located on opposite alleles (in trans); therefore, the PM3 criterion cannot be applied. In summary, the c.837delT variant meets PVS1 + PM2, which supports a classification of likely pathogenic. Further functional studies should be conducted to validate their pathogenicity.

Wilson disease has several hotspot variants that vary significantly across different populations. The most common variant in European Wilson disease patients is p.His1069Gln, with a frequency ranging from 13 to 61% (22). In Asian populations, the prevalent variant is p.Arg778Leu, which is found in 34–38% of cases. Among Chinese patients with Wilson disease, three high-frequency pathogenic variants, p.Arg778Leu, p.Pro992Leu, and p.Thr935Met, account for 50–60% of all pathogenic variants (23). A study involving 196 Chinese Wilson disease patients undergoing genetic testing revealed the highest mutation rates in exons 8, 13, and 16 (24). A meta-analysis revealed that exon 8 had the greatest variety of variants, followed by exons 18, 2, and 14 (25). The compound heterozygous variant in our patient was not located in the common hotspot exons 8, 13, or 16 in the Chinese population.

The most common clinical manifestations of Wilson disease can be categorized into three patterns: hepatic, neurological, and psychiatric. Liver damage typically occurs within the first decade of life, while neuropsychiatric symptoms more commonly appear in the second or third decade (26). The patient initially presented with elevated liver enzyme levels and cirrhosis, which later developed tremors and dystonia. She exhibited classic *risus sardonicus*, characterized by involuntary grimacing, mouth opening, and upper lip retraction. Both hepatic and neurological symptoms were prominent in this patient. Notably, previous reports have indicated that Kayser–Fleischer rings are present in nearly all patients with neurologic Wilson disease at the time of presentation (27). In our patient, the Kayser–Fleischer ring was not detected on initial gross inspection but was identified as positive upon formal slit-lamp examination, underscoring the necessity of performing a formal slit-lamp evaluation for Kayser–Fleischer rings.

Previous studies have indicated that a ceruloplasmin level below 0.1 g/L strongly supports the diagnosis of Wilson disease (28). The proband’s ceruloplasmin level at initial presentation was only 0.04 g/L, which was highly suggestive and necessitated the exclusion of Wilson disease. However, according to previous reports, ceruloplasmin levels do not necessarily correlate with the disease severity. For instance, Chappuis et al. (29) reported a patient with Wilson disease who had a ceruloplasmin level of 0.03 g/L and a significant splice-site variant who presented with only mild hepatic and neurological symptoms.

Wilson disease is rare and characterized by numerous types of variants, often occurring in a compound heterozygous state, which makes genotype–phenotype associations difficult. Previous studies have reported clinical heterogeneity even among three patients carrying the same homozygous variant (two with hepatic phenotype and one with neurological presentation), suggesting that other factors may also contribute to phenotypic expression (30). Earlier research has also found that patients carrying one or two deletion/nonsense variant alleles exhibit more severe symptoms and an earlier age of onset, compared with those carrying two missense variant alleles, although no correlation was observed between variant type and the initial manifestation of Wilson disease (31). Our patient presented with both significant hepatic and neurological symptoms, which may be related to the presence of a deletion variant. The other variant in this patient is a splice mutation, c.1543+40G>A, for which we did not find any clinical phenotypic data. However, we identified reports on the phenotype associated with a closely located variant, c.1543+1G>C, in the homozygous state. The c.1543+1G>C variant alters the first conserved nucleotide of the donor splice site, likely leading to exon skipping or the use of an alternative splice site. Two patients homozygous for c.1543+1G>C developed severe neurological disease between 13 and 15 years of age, manifesting as dysarthria, dysphagia, and tremors, followed by acute hepatitis (32). Notably, in the patient we report, one variant is located in exon 2, while the other is predicted to lead to skipping of exon 3. Although the patient’s genotype is compound heterozygous, the protein domains affected by these two distinct variants are located close to each other. Whether the clinical presentation of such patients differs from that of compound heterozygotes with variants located farther apart warrants attention in future genotype–phenotype studies of Wilson disease.

The patient initially presented to the hepatology clinic because of abnormal liver function and cirrhosis discovered during routine medical examinations. Her parents were non-consanguineous and she denied any relevant family history. At that time, the consulting physician did not immediately consider the diagnosis of Wilson disease and did not recommend genetic testing. After developing neurological signs of lower limb tremor, the patient subsequently presented to the neurology department. Brain magnetic resonance imaging (MRI) revealed characteristic abnormal intracranial signals, leading to comprehensive genetic testing and a definitive diagnosis. The global carrier frequency for *ATP7B* variants is approximately 1 in 90 (33, 34). The patient’s mother was a heterozygous carrier of a point variant, while the patient herself carried a *novel* variant, resulting in a disease due to compound heterozygous variants. However, it should be noted that although the patient’s parents acknowledge their biological relationship with the patient, STR analysis has not been performed, and the possibility of parental germline mosaicism cannot be excluded; therefore, the variant in the patient may not necessarily be *de novo*. Furthermore, the lack of genetic testing results from the patient’s siblings may limit the strength of evidence regarding the pathogenicity of the proband’s genetic variant. We will continue to monitor the patient’s family over follow-up, particularly observing whether the brother and sister develop clinical symptoms, and if necessary, we will recommend *ATP7B* genetic testing for them as well.

The patient initially received penicillamine, but it was stopped due to a rash before the dose could be escalated. She was then switched to intravenous dimercaptopropanesulfonate (DMPS) for copper chelation, along with oral zinc therapy. A total of 11 courses of DMPS were administered over 3 months (each course consisted of daily administration for five consecutive days, followed by a 2-day interval). The 24-h urinary copper excretion ranged from 667.91 to 1,519.35 μg/24 h, reaching a peak on day 6 of treatment and gradually declining thereafter. After discharge, the patient was switched to oral dimercaptosuccinic acid (DMSA). DMSA is recommended for the treatment of Wilson disease in the Chinese Guidelines for the Diagnosis and Treatment of Wilson Disease (35). Although trientine is considered more standard in many international guidelines, it is expensive and only became available in China in 2023, with limited accessibility at present. Following 3 months of treatment, her dysarthria showed a slight worsening. Liver function tests revealed normal levels of alanine aminotransferase (ALT), aspartate aminotransferase (AST), and gamma-glutamyl transferase (GGT), while total bilirubin (TBIL) and direct bilirubin (DBIL) were mildly elevated. At the 6-month follow-up, the patient’s liver function remained stable, and there was no further progression of neurological symptoms.

Reviewing the diagnostic and treatment process of this patient, the initial diagnosis was delayed. In young patients with unexplained cirrhosis, screening for Wilson disease should not be overlooked even in the absence of a relevant family history or consanguineous marriage, because most patients with Wilson disease are compound heterozygotes (36), and consanguineous marriage is not a prerequisite. The patient presented with both cirrhosis and neurological symptoms, requiring particular caution in initial treatment, as the use of chelating agents may cause further worsening of neurological function, especially in patients with pre-existing neurological symptoms. This patient was initiated on a low-dose chelating agent to mitigate this risk.

Wilson disease is one of the few treatable inherited disorders, making early diagnosis and treatment crucial. Timely intervention can prevent irreversible tissue and organ damage. Therefore, prompt and accurate genetic testing is essential for facilitating patient recovery. This study identified a novel deletion variant in the *ATP7B* gene and described the patient’s phenotype, which enriches the pathogenic gene spectrum of the *ATP7B* gene.

## Data Availability

The datasets presented in this study can be found in online repositories. The names of the repository/repositories and accession number(s) can be found in the article/supplementary material.

## Footnotes

1
http://www.wilsondisease.med.ualberta.ca/database.asp

## References

[1] Wungjiranirun M Sharzehi K. Wilson's disease. Semin Neurol. (2023) 43:626–33. doi: 10.1055/s-0043-1771465, 37607588

[2] Diao S Lǚ C Huang Y Zhou Z Liu A Hong M. Linear structural features of Wilson's disease and its correlation with neurological symptoms. Medicine (Baltimore). (2022) 101:e31386. doi: 10.1097/MD.0000000000031386, 36550817 PMC9771331

[3] Rędzia-Ogrodnik B Członkowska A Antos A Bembenek J Kurkowska-Jastrzębska I Przybyłkowski A . Pathognomonic neuroradiological signs in Wilson's disease – truth or myth? Parkinsonism Relat Disord. (2023) 107:105247. doi: 10.1016/j.parkreldis.2022.105247, 36543734

[4] Stenson PD Mort M Ball EV Shaw K Phillips A Cooper DN. The human gene mutation database: building a comprehensive mutation repository for clinical and molecular genetics, diagnostic testing and personalized genomic medicine. Hum Genet. (2014) 133:1–9. doi: 10.1007/s00439-013-1358-4, 24077912 PMC3898141

[5] Mameli E Lepori MB Chiappe F Ranucci G di Dato F Iorio R . Wilson's disease caused by alternative splicing and Alu exonization due to a homozygous 3039-bp deletion spanning from intron 1 to exon 2 of the ATP7B gene. Gene. (2015) 569:276–9. doi: 10.1016/j.gene.2015.05.067, 26031236

[6] Zhou Z Zhang S Bi Y Duan W Gao H. A novel mutation in the ATP7B gene causing hepatolenticular degeneration in a Chinese family: a case report. Medicine. (2024) 103:e38849. doi: 10.1097/MD.0000000000038849, 39093796 PMC11296479

[7] Liu G Ma D Cheng J Zhang J Luo C Sun Y . Identification and characterization of a novel 43-bp deletion mutation of the ATP7B gene in a Chinese patient with Wilson's disease: a case report. BMC Med Genet. (2018) 19:61. doi: 10.1186/s12881-018-0567-z, 29649982 PMC5898064

[8] Tanzi RE Petrukhin K Chernov I Pellequer JL Wasco W Ross B . The Wilson disease gene is a copper transporting ATPase with homology to the Menkes disease gene. Nat Genet. (1993) 5:344–50. doi: 10.1038/ng1293-344, 8298641

[9] Bull PC Thomas GR Rommens JM Forbes JR Cox DW. The Wilson disease gene is a putative copper transporting P-type ATPase similar to the Menkes gene. Nat Genet. (1993) 5:327–37. doi: 10.1038/ng1293-327, 8298639

[10] Gourdon P Liu X Skjørringe T Liu X-Y Morth JP Møller LB . Crystal structure of a copper-transporting PIB-type ATPase. Nature. (2011) 475:59–64. doi: 10.1038/nature10191, 21716286

[11] Cater MA La Fontaine S Mercer JFB. Copper binding to the N-terminal metal-binding sites or the CPC motif is not essential for copper-induced trafficking of the human Wilson protein (ATP7B). Biochem J. (2007) 401:143–53. doi: 10.1042/BJ20061055, 16939419 PMC1698686

[12] Cater MA Forbes J La Fontaine S Cox D Mercer JFB. Intracellular trafficking of the human Wilson protein: the role of the six N-terminal metal-binding sites. Biochem J. (2004) 380:805–13. doi: 10.1042/bj20031804, 14998371 PMC1224206

[13] Lenartowicz MG Krzeptowski W. Structure and function of ATP7A and ATP7B proteins--cu-transporting ATPases. Postepy Biochem. (2010) 56:317–27. 21117320

[14] Calvo JS Heger T Kabin E Mowrey WR del Angel G Ding W . Functional screen of Wilson disease ATP7B variants reveals residual transport activities. Hum Mutat. (2025) 2025:7485658. doi: 10.1155/humu/7485658, 40661833 PMC12259332

[15] Fatemi N Sarkar B. Molecular mechanism of copper transport in Wilson disease. Environ Health Persp. (2002) 110:695–8. doi: 10.1289/ehp.02110s5695, 12426114 PMC1241227

[16] Sazinsky MH Mandal AK Argüello JM Rosenzweig AC. Structure of the ATP binding domain from the *Archaeoglobus fulgidus* cu+-ATPase. J Biol Chem. (2006) 281:11161–6. doi: 10.1074/jbc.M510708200, 16495228

[17] Zhang T Song W Mao Z. Phenotypic and genetic characterization of children with Wilson disease from Northeast China. BMC Pediatr. (2024) 24:576. doi: 10.1186/s12887-024-05045-x, 39267050 PMC11391784

[18] Xu W Wang R Dong Y Wu Z. Pathogenicity of Intronic and synonymous variants of ATP7B in Wilson disease. J Mol Diagn: JMD. (2023) 25:57–67. doi: 10.1016/j.jmoldx.2022.10.002, 36343861

[19] Huang C Fang M Xiao X Gao Z Wang Y Gao C. Genetic studies discover novel coding and non-coding mutations in patients with Wilson's disease in China. J Clin Lab Anal. (2022) 36:e24459. doi: 10.1002/jcla.24459, 35470480 PMC9169201

[20] Lutsenko S Petrukhin K Cooper MJ Gilliam CT Kaplan JH. N-terminal domains of human copper-transporting adenosine triphosphatases (the Wilson's and Menkes disease proteins) bind copper selectively in vivo and in vitro with stoichiometry of one copper per metal-binding repeat. J Biol Chem. (1997) 272:18939–44. doi: 10.1074/jbc.272.30.18939, 9228074

[21] Richards S Aziz N Bale S Bick D das S Gastier-Foster J . Standards and guidelines for the interpretation of sequence variants: a joint consensus recommendation of the American College of Medical Genetics and Genomics and the Association for Molecular Pathology. Genet Med. (2015) 17:405–24. doi: 10.1038/gim.2015.30, 25741868 PMC4544753

[22] Bennett J Hahn SH. Clinical molecular diagnosis of Wilson disease. Semin Liver Dis. (2011) 31:233–8. doi: 10.1055/s-0031-1286054, 21901653

[23] Cheng N Wang H Wu W Yang R Liu L Han Y . Spectrum of ATP7B mutations and genotype-phenotype correlation in large-scale Chinese patients with Wilson disease. Clin Genet. (2017) 92:69–79. doi: 10.1111/cge.12951, 27982432

[24] Li M Ma J Wang W Yang X Luo K. Mutation analysis of the ATP7B gene and genotype-phenotype correlation in Chinese patients with Wilson disease. BMC Gastroenterol. (2021) 21:339. doi: 10.1186/s12876-021-01911-5, 34470610 PMC8411542

[25] Lao TD Le TAH. Systematic analysis and insights into the mutation Spectrum and ethnic differences in *ATP7B* mutations associated with Wilson disease. Biomark Insights. (2024) 19:11772719241297169. doi: 10.1177/11772719241297169, 39502306 PMC11536366

[26] Machado A Chien HF Deguti MM Fen Chien H Mitiko Deguti M Cançado E . Neurological manifestations in Wilson's disease: report of 119 cases. Mov Disord. (2006) 21:2192–6. doi: 10.1002/mds.21170, 17078070

[27] Demirkiran M Jankovic J Lewis RA Cox DW. Neurologic presentation of Wilson disease without Kayser-Fleischer rings. Neurology. (1996) 46:1040–3. doi: 10.1212/WNL.46.4.1040, 8780087

[28] Ryan A Nevitt SJ Tuohy O Cook P. Biomarkers for diagnosis of Wilson's disease. Cochrane Database Syst Rev. (2019) 2019:D12267. doi: 10.1002/14651858.CD012267 PMC6953362 31743430

[29] Chappuis P Callebert J Quignon V Woimant F Laplanche J. Late neurological presentations of Wilson disease patients in French population and identification of 8 novel mutations in the ATP7B gene. J Trace Elements Med Biol. (2007) 21:37–42. doi: 10.1016/j.jtemb.2006.11.002, 17317524

[30] Kumari N Kumar A Thapa BR Modi M Pal A Prasad R. Characterization of mutation spectrum and identification of novel mutations in ATP7B gene from a cohort of Wilson disease patients: functional and therapeutic implications. Hum Mutat. (2018) 39:1926–41. doi: 10.1002/humu.23614, 30120852

[31] Gromadzka G Schmidt HH Genschel J Schmidt HH‐J Bochow B Rodo M . Frameshift and nonsense mutations in the gene for ATPase7B are associated with severe impairment of copper metabolism and with an early clinical manifestation of Wilson's disease. Clin Genet. (2005) 68:524–32. doi: 10.1111/j.1399-0004.2005.00528.x, 16283883

[32] Deguti MM Genschel J Cancado ELR Barbosa ER Bochow B Mucenic M . Wilson disease: novel mutations in the ATP7B gene and clinical correlation in Brazilian patients. Hum Mutat. (2004) 23:398. doi: 10.1002/humu.9227, 15024742

[33] Olsson C Waldenström ME Westermark K Landegren U Syvänen AC. Determination of the frequencies of ten allelic variants of the Wilson disease gene (*ATP7B*), in pooled DNA samples. Eur J Hum Genet. (2000) 8:933–8. doi: 10.1038/sj.ejhg.5200566, 11175281

[34] Sandahl TD Laursen TL Munk DE Vilstrup H Weiss KH Ott P. The prevalence of Wilson's disease: an update. Hepatology. (2020) 71:722–32. doi: 10.1002/hep.30911, 31449670

[35] Inherited Metabolic Liver Disease Collaboration Group CSOH. Guidelines for the diagnosis and treatment of hepatolenticular degeneration (2022 edition). Chin J Hepatol. (2022) 30:9–20. doi: 10.3760/cma.j.cn501113-20211217-00603 PMC12770193 35152665

[36] Schilsky ML Roberts EA Bronstein JM Dhawan A Hamilton JP Rivard AM . A multidisciplinary approach to the diagnosis and management of Wilson disease: executive summary of the 2022 practice guidance on Wilson disease from the American Association for the Study of Liver Diseases. Hepatology. (2022) 77:1428–55. doi: 10.1002/hep.32805, 36152019

